# Treatment of humeral shaft nonunion by exchange intramedullary nailing and circumferential cortical strut allograft: the Sarcophagus technique

**DOI:** 10.1016/j.xrrt.2026.100768

**Published:** 2026-05-05

**Authors:** Alexander J. Vervaecke, Romain Chevallier, Victor Housset, Thomas Bauer, Jean-David Werthel

**Affiliations:** a Hôpital Ambroise-Paré, Boulogne Billancourt, France; b Orthopaedic Center Antwerp, Antwerp, Belgium; c L'Institut de Chirurgie du Sport et de l’Arthrose (ICSA), Paris, France; d Clinique Maussins Nollet, Paris, France

**Keywords:** Shoulder surgery, Humerus fracture, Humeral osteosynthesis, Nonunion, Upper limb trauma, Intramedullary nail, Strut graft, Humeral shaft

## Abstract

**Background:**

Nonunion of the humeral shaft presents a complex surgical challenge, with ongoing debate surrounding optimal fixation and grafting strategies. This study evaluates the clinical and radiographic outcomes of a novel surgical approach, intramedullary nailing combined with circumferential (360°) cortical strut allografting, termed the Sarcophagus technique. We hypothesized that the Sarcophagus technique would be a safe and effective treatment strategy for humeral shaft nonunion, offering union rates comparable to conventional techniques.

**Methods:**

We conducted a retrospective case series of 8 patients with aseptic, atrophic humeral shaft nonunion treated using the Sarcophagus technique between 2017 and 2021. All patients had previously undergone intramedullary nailing as the index treatment. The primary outcome was radiographic union at 12 months. Secondary outcomes included time to union, complication rates, and functional scores (American Shoulder and Elbow Surgeons, Simple Shoulder Test, Quick Disabilities of the Arm, Shoulder, and Hand, and Auto-Constant) assessed at 3, 6, and 12 months.

**Results:**

Radiographic union was achieved in 7 of 8 patients (87.5%), with a mean time to union of 5.8 ± 1.3 months. Functional outcomes demonstrated progressive improvement across all measures during follow-up. No post-operative infections occurred. One patient had persistent fracture nonunion but remained minimally symptomatic and declined re-revision. Transient radial nerve palsy occurred in 1 patient (12.5%) and resolved spontaneously within 3 months.

**Conclusion:**

The Sarcophagus technique demonstrated a high union rate and favorable functional recovery in patients with humeral shaft nonunion. Larger comparative studies are warranted to further establish the role of this technique in complex humeral nonunions.

Humeral shaft fractures represent approximately 3% of all fractures, with an incidence of 1 per 13,000 individuals.^3^ These injuries rank third among diaphyseal fractures, after those of the femur and tibia.^14^ While conservative management using functional bracing is common and generally effective, with union rates ranging from 70% to 87%, surgical intervention may be required in displaced or unstable fractures.^18^^,^^22^ Operative options include open reduction and internal fixation with plating or intramedullary (IM) nailing.^23^ However, no clear consensus exists regarding the superior technique in terms of union rates, time to healing, functional recovery, infection risk, or incidence of radial nerve injury.^1^^,^^4^^,^^21^

Despite the relatively high incidence of humeral shaft fractures, the surgical treatment of associated nonunion remains less well established. Nonunion is frequently associated with pain, functional impairment, and the need for more complex procedures.^2^ In such cases, depending on the type of nonunion, treatment often involves achieving stable fixation by plating or nailing, combined with autologous iliac crest bone grafting.^11^ Reported union rates vary from 60% to 100% in literature, depending on patient characteristics and the utilized surgical technique.^11^^,^^12^^,^^15^

The use of autografts is the gold standard due to their osteogenic, osteoinductive, and osteoconductive properties.^19^ However, autograft harvesting carries the risk of donor site morbidity, and graft volume may be limited, particularly in patients who have undergone prior harvests. In contrast, allograft eliminates donor site morbidity and provides a practical alternative with added structural support, albeit with reduced osteogenic potential.^8^ Cortical strut allografts have been used to augment fixation in long bone reconstruction; the technique was first described by Hornicek et al and later expanded on under the term of the “sandwich technique.”^7^^,^^9^ In these constructs, cortical struts are typically applied along 1 or 2 cortices to augment plate fixation, providing additional structural support and biological scaffolding. To our knowledge, however, the use of circumferential (360°) cortical strut grafting in combination with exchange IM nailing has not been previously described. We refer to this construct as the Sarcophagus technique, in which multiple cortical struts encase the nonunion site, providing both mechanical support and an osteoconductive scaffold.

The primary aim of this study was to evaluate the radiographic union rate and safety profile of the Sarcophagus technique in the treatment of humeral shaft nonunion. Secondary aims included the assessment of functional outcomes at 3, 6, and 12 months and an analysis of complications. We hypothesized that the Sarcophagus technique would be a safe and effective strategy, offering union rates comparable to conventional techniques.

## Patients and methods

### Study design

This was a retrospective, single-center, multisurgeon case series of patients who underwent surgical treatment for aseptic humeral shaft nonunion using the Sarcophagus technique between January 2017 and September 2021. The technique consists of IM nailing combined with circumferential (360°) cortical strut allografting. Inclusion criteria were (1) aseptic nonunion, both primary or after failed previous surgical treatment, defined as absence of clinical or radiological healing ≥9 months after the index procedure, no signs of healing progression over the previous 3 months, and absence of infection as verified by negative serum inflammatory markers (C-reactive protein, erythrocyte sedimentation rate, leukocytosis) and absence of sinus tract or wound drainage; (2) atrophic nonunion, based on radiographic appearance with lack of callous formation; and (3) minimum follow-up of 12 months after the revision surgery. Demographic data included age, sex, smoking status, body mass index, relevant comorbidities, and prior surgical history. A total of 9 patients were eligible; 1 was lost to follow-up, leaving 8 patients for analysis. All included patients had previously undergone IM nailing as their primary surgical treatment. Informed consent was obtained from all individual participants included in the study, and institutional review board approval was granted by the local ethics committee (No. 00010835).

### Surgical procedure

All procedures were performed under general anesthesia with standard antibiotic prophylaxis. Patients were positioned supine with the affected arm placed on a radiolucent arm table. No tourniquet was used. A direct lateral approach to the humeral shaft was employed, and the radial nerve was routinely identified at the intermuscular septum and carefully exposed and neurolyzed across the fracture zone. The original proximal surgical incision was systematically reused and corresponded to an anterolateral approach in all cases. Previously implanted hardware was removed through existing incisions.

The nonunion site was thoroughly débrided of fibrous tissue and avascular bone until healthy, bleeding bone edges were exposed. Cortical notches were created at the fracture edges using a bone chisel to facilitate revascularization and graft incorporation. A locked humeral IM nail (T2 Humeral Nail, Stryker, Kalamazoo, MI, USA) was inserted after manual canal reaming, with a diameter 1 mm larger than the original implant. Rotational alignment was verified intraoperatively using the contralateral arm as a reference, and reduction was confirmed under fluoroscopy. Proximal locking was performed using 2 multiplanar interlocking screws. Distal locking was achieved using 1 or 2 uniplanar interlocking screws depending on the intraoperative stability of the construct to ensure adequate rotational and axial stability.

Each patient received a frozen tibial shaft allograft, thawed, and soaked in rifampicin for 20 minutes. Cortical struts were shaped to approximately 12 mm in width and 9–11 cm in length depending on the fracture geometry. Strut length was chosen as the maximal length that could be safely accommodated: proximally, the graft was positioned without compromising the insertions of the deltoid, pectoralis major, latissimus dorsi, or teres major, while distally it was stopped proximally to the metaphyseal flare of the humerus. If no prior iliac crest graft had been harvested, autologous spongious bone was collected and packed into the nonunion site. Otherwise, additional cancellous allograft was used.

**Figure 1:**
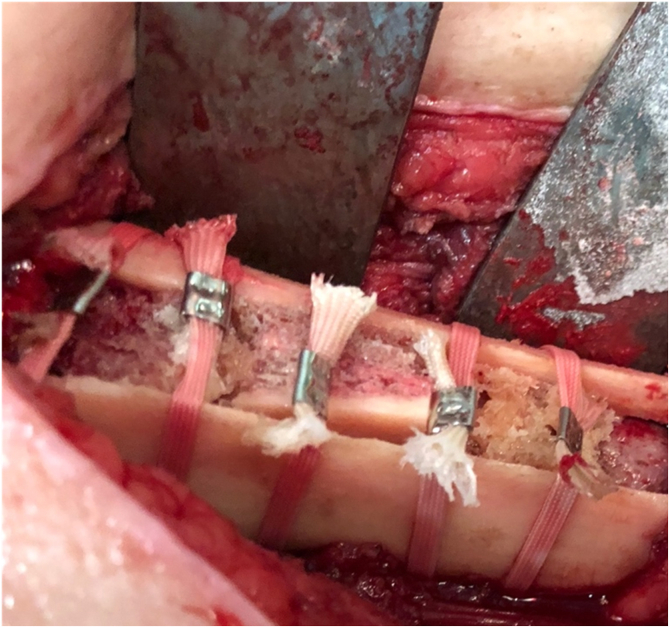
Intraoperative image of the Sarcophagus technique. Circumferential cortical strut allografts are secured around the humeral shaft using cerclage tapes, with impacted cancellous autograft from the iliac crest filling the nonunion site. This configuration creates a mechanically supportive and biologically active environment to promote fracture union.

Cerclage tapes (Strapflex, FH Ortho, France) were passed loosely around the humeral shaft, ensuring the radial nerve was protected at all times. Two to 3 cortical struts were placed circumferentially, at least 2 diametrically opposed, around the diaphysis to encase the nonunion site. Cerclage tapes were then tightened to secure the construct, completing the Sarcophagus configuration (Fig. 1). The wound was closed in layers.

The arm was immobilized in a sling for 6 weeks. Passive and pendular shoulder motion, as well as full elbow range of motion, were initiated immediately post-operatively. Active rehabilitation began at 6 weeks.

### Post-operative evaluation

Patients were followed at regular intervals with clinical and radiographic evaluation (Fig. 1). The primary outcome was radiographic union, defined as bridging callus across at least 2 cortices on orthogonal views. The time to union was also recorded.

Secondary outcomes included functional assessment using the following validated scores: American Shoulder and Elbow Surgeons score, Simple Shoulder Test, Quick Disabilities of the Arm, Shoulder, and Hand (QuickDASH), and Auto-Constant Score (a self-administered version of the Constant Score). These scores were collected at 3 months, 6 months, and 1 year post-operatively using patient-completed questionnaires during routine clinic visits.

### Statistical analysis

Statistical analysis was limited to a descriptive approach due to the small cohort size. Continuous variables are presented as mean values with the standard deviation for time to union and corresponding 95% confidence intervals (CIs) for outcome scores. Categorical variables are presented as frequencies and percentages. Functional outcome scores were recorded to illustrate clinical progression during follow-up. Given the limited sample size (n = 8), formal hypothesis testing was not performed. Statistical summaries were performed using Microsoft Excel.

## Results

### Patient characteristics

Eight patients met the inclusion criteria during the study period. Seven were female (87.5%), with a mean age of 57.8 ± 19.4 years. Two patients (25%) were active smokers at the time of surgery, and the mean body mass index was 24.6 ± 3.2 kg/m^2^. All patients had been previously treated with IM nailing, and all cases represented secondary aseptic nonunion after initial intervention.

### Radiographic union

**Figure 2:**
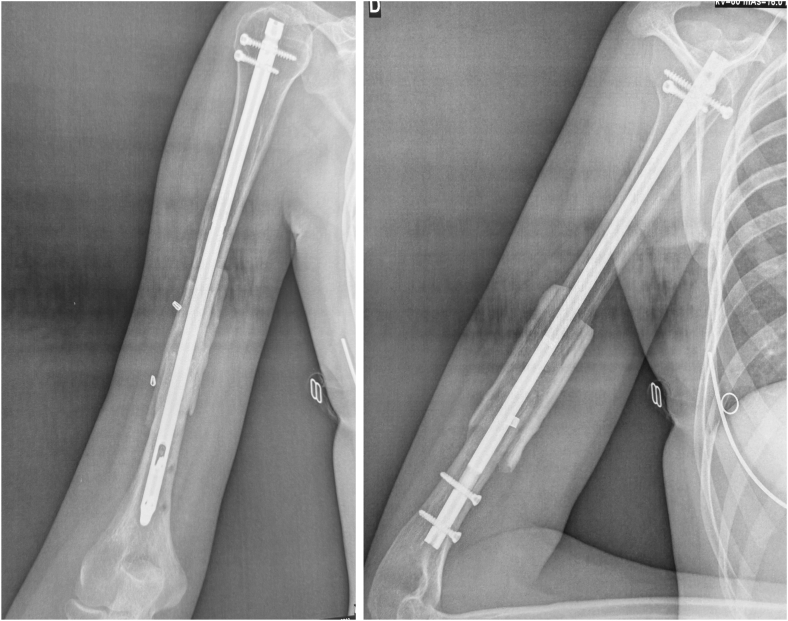
Anteroposterior and lateral radiographs of the humerus obtained at 3-month follow-up demonstrating early allograft integration following treatment of a humeral shaft nonunion with exchange intramedullary nailing and circumferential cortical strut allograft augmentation (Sarcophagus technique).

**Figure 3:**
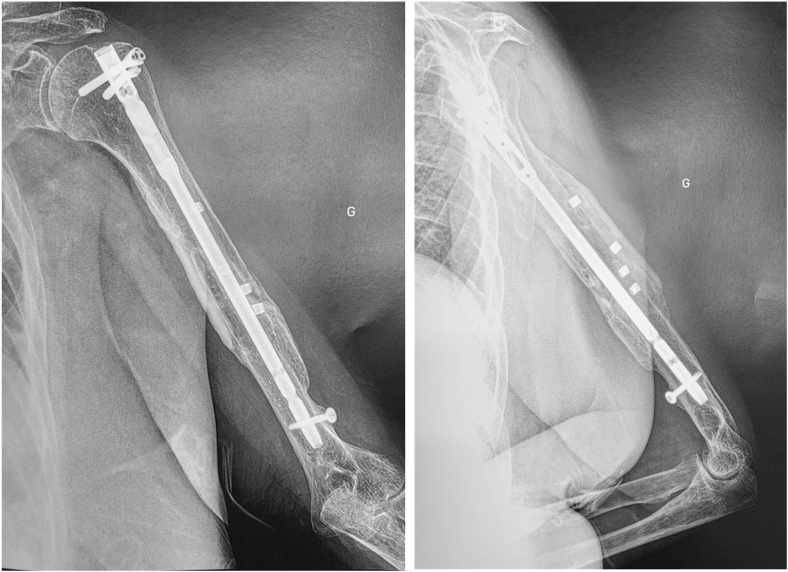
Anteroposterior and lateral radiographs at 12 months post-operatively demonstrating complete fracture consolidation and bony incorporation of circumferential cortical strut allografts following treatment of humeral shaft nonunion with exchange intramedullary nailing.

**Figure 4:**
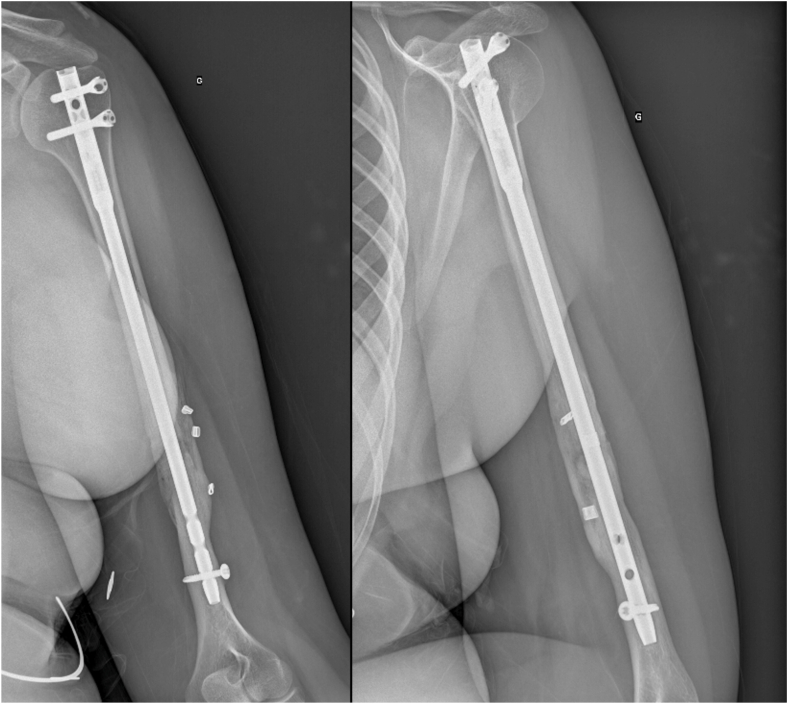
Anteroposterior and lateral radiographs at 7 months post-operatively demonstrate fracture union and graft incorporation.

At 12 months post-operatively, 7 out of 8 patients (87.5%) demonstrated radiological union based on standard radiographic assessment (Fig. 2). The mean time to union in those 7 patients was 5.8 ± 1.3 months (range: 4.0–9.3; 95% CI: 4.6–7.0 months) (Figure 3, Figure 4 and Figure 3, Figure 4). One patient experienced recurrent fracture nonunion with distal screw breakage of the IM nail at one year post-operatively.

### Functional assessment

Functional outcome scores increased over the follow-up period (Table I). Between 3 months and 12 months post-operatively, the mean American Shoulder and Elbow Surgeons score increased from 42.1 to 57.6 (+36.8%), the Simple Shoulder Test from 26.0% to 61.5% (+136.5%), and the Auto-Constant Score from 46.0% to 80.0% (+73.9%). Over the same period, the QuickDASH score decreased from 65.0% to 28.1%, corresponding to a 56.8% improvement. The single patient with persistent nonunion demonstrated stable function and did not elect for further surgical intervention.

**Table I:** Evolution of functional outcome scores during follow-up after treatment of humeral shaft nonunion with the Sarcophagus technique.

Functional score	3 mo	6 mo	12 mo
ASES (points)	42.1 (95% CI: 37.8–46.4)	53.5 (95% CI: 49.2–57.8)	57.6 (95% CI: 53.3 – 61.9)
SST (%)	26.0 (95% CI: 21.0–31.0)	48.9 (95% CI: 43.9–53.9)	61.5 (95% CI: 56.5 – 66.3)
Auto-Constant (%)	46.0 (95% CI: 42.7–49.3)	63.0 (95% CI: 59.7–66.3)	80.0 (95% CI: 76.7 – 83.3)
QuickDASH (%)	65.0 (95% CI: 59.3–70.7)	44.0 (95% CI: 38.3–49.7)	28.1 (95% CI: 22.4 – 33.8)

*ASES*, American Shoulder and Elbow Surgeons; *SST*, Simple Shoulder Test; *QuickDASH*, Quick Disabilities of the Arm, Shoulder, and Hand.

Values represent mean scores with corresponding 95% confidence intervals (CIs) at each follow-up time point (3, 6, and 12 months). The results are presented descriptively to illustrate the clinical evolution of functional outcomes during follow-up.

### Complications

One patient (12.5%) developed a transient radial nerve palsy, which resolved completely within 3 months without intervention. No superficial or deep infections were reported. Two patients (25.0%) underwent elective hardware removal beyond the 1-year mark due to implant-related discomfort after confirmed fracture union.

## Discussion

This study demonstrates that humeral shaft nonunion treated with exchange IM nailing and circumferential cortical strut grafting achieved a radiographic union rate of 87.5% at 12 months post-operatively, with a mean time to union of 5.8 months. Functional outcomes improved progressively throughout the follow-up period. These findings fall within the union rates reported for humeral shaft nonunion in the literature (60–100%), depending on surgical strategy and patient population.^2^^,^^9^^,^^10^ While cortical strut grafts have previously been described as augmentation techniques in combination with plate fixation, typically applied along 1 or 2 cortices, the Sarcophagus technique differs by combining IM fixation with circumferential (360°) cortical strut grafting. In this configuration, multiple cortical struts are arranged around the humeral shaft to encase the nonunion site, creating a combined mechanical and biological scaffold for bone healing.

### Union rate

Previous studies have documented a wide spectrum of outcomes after surgical treatment of humeral nonunions with different fixation and grafting techniques. Chantelot et al reported a 95% union rate within 4 to 6 months in a series of 21 patients treated with either plate or nail fixation.^5^ Similarly, Segonds et al observed 100% union in 30 patients managed with plate fixation combined with synthetic bone grafting, although the follow-up duration was not clearly specified.^20^ In contrast, Ilyas et al achieved only a 60% union rate at 6 months following IM nailing with iliac crest autograft, which may reflect the inclusion of patients with open fractures at initial injury.^10^ Martínez et al compared plating and nailing (both with autograft) and found slightly faster union in the nail group (4.2 vs. 4.7 months) but with 100% union in both cohorts.^11^ Additionally, a systematic review encompassing 164 patients with humeral shaft nonunion treated with IM nailing and autologous bone graft reported union rates ranging from 56% to 100%, with a pooled mean of 88%.^15^ Our series adds to this body of evidence by specifically evaluating post-operative humeral nonunions treated with circumferential cortical allografts in combination with exchange IM nailing.

### Functional outcome and graft comparison

Progressive improvement in all functional scores was observed, with a mean QuickDASH improvement of 35 points from 3 to 12 months. This compares favorably with the findings of Padhye et al, who reported a 30-point DASH improvement at 16 months in a heterogeneous cohort treated with plates, nails, fibular strut grafts, or external fixation.^13^ Their best results were seen in patients treated with plate fixation and cancellous autograft.

Unlike the IM placement of fibular grafts in Padhye's study, our technique employs extramedullary tibial cortical strut allograft arranged circumferentially around the humeral shaft, forming a biological encasement of the nonunion site. While autografts remain the gold standard due to their osteogenic, osteoinductive, and osteoconductive properties, their use may be limited by donor site morbidity and graft availability.^8^^,^^19^ Ideally, both graft types can be combined, with autograft providing biological activity, and cortical allograft offering additional structural and osteoconductive support. In our series, cortical strut allografts were combined with cancellous allograft in patients with comorbidities, prior iliac crest harvest or compromised bone quality, otherwise, autograft was preferred.^6^

Cortical strut grafts have also been described as biological and structural augmentation in combination with plate fixation for the treatment of humeral nonunions and bone loss.^9^ Several studies have reported satisfactory union rates using plate constructs reinforced with cortical strut grafts, highlighting the importance of combining mechanical stabilization with biological augmentation in complex reconstructions.^16^^,^^17^

### Radial nerve complications

One patient (12.5%) experienced transient radial nerve palsy which was resolved at 3 months post-operatively. While this incidence appears higher than the 0–6.2% rate reported in the literature, the smaller sample size in our study may limit the ability to deduct definitive conclusions.^15^ The etiology for this complication may be multifactorial, involving iatrogenic nerve traction or compression from dissection, cerclage placement, or bulk from the cortical struts. Given this risk, intraoperative neuromonitoring, careful graft sizing, and alternative graft configurations may help minimize nerve tension and reduce the incidence of iatrogenic palsy.

### Study limitations

This study has several limitations. First, the sample size was small (n = 8), which limits statistical power and restricts the generalizability of the findings. Due to the limited cohort size, the statistical analysis was restricted to a descriptive approach and formal hypothesis testing was not performed. In addition, the small cohort may amplify the apparent complication rate, particularly regarding the observed radial nerve palsy (12.5%). However, considering the rarity of post-operative humeral shaft nonunion, even small cohorts contribute meaningful data. Second, although the 12-month follow-up period is sufficient to evaluate fracture union, longer-term outcomes such as hardware failure, refracture, or late complications remain unknown. Third, the retrospective nature of the study introduces inherent limitations, including potential selection bias and lack of standardized pre-operative functional assessments. Lastly, the absence of a control group prevents direct comparison with other established techniques such as plating with graft augmentation or alternative grafting techniques.

## Conclusion

This case series introduces a novel surgical approach for treating humeral shaft nonunion, combining IM nailing with circumferential cortical allografting. The method demonstrated a high rate of radiographic union and favorable functional outcomes. These promising results support further investigation in larger, controlled studies to establish the technique's role in the management of complex humeral nonunions.

## Disclaimers:

Funding: No funding was disclosed by the authors.

Conflicts of interest: Jean-David Werthel serves as a consultant for Stryker and receives royalties for shoulder arthroplasty implants from Stryker. Any additional authors, their immediate families, and any research foundations with which they are affiliated have not received any financial payments or other benefits from any commercial entity related to the subject of this article.
